# Perspectives on Non-BLT Humanized Mouse Models for Studying HIV Pathogenesis and Therapy

**DOI:** 10.3390/v13050776

**Published:** 2021-04-28

**Authors:** Kazutaka Terahara, Ryutaro Iwabuchi, Yasuko Tsunetsugu-Yokota

**Affiliations:** 1 Research Center for Drug and Vaccine Development, National Institute of Infectious Diseases, Tokyo 162-8640, Japan; tera@nih.go.jp (K.T.); buchitaro@toki.waseda.jp (R.I.); 2 Department of Life Science and Medical Bioscience, Waseda University, Tokyo 162-8480, Japan; 3 Department of Medical Technology, School of Human Sciences, Tokyo University of Technology, Tokyo 144-8535, Japan

**Keywords:** humanized mice, non-BLT, immunological features, HIV infection, pathogenesis, therapy

## Abstract

A variety of humanized mice, which are reconstituted only with human hematopoietic stem cells (HSC) or with fetal thymus and HSCs, have been developed and widely utilized as in vivo animal models of HIV-1 infection. The models represent some aspects of HIV-mediated pathogenesis in humans and are useful for the evaluation of therapeutic regimens. However, there are several limitations in these models, including their incomplete immune responses and poor distribution of human cells to the secondary lymphoid tissues. These limitations are common in many humanized mouse models and are critical issues that need to be addressed. As distinct defects exist in each model, we need to be cautious about the experimental design and interpretation of the outcomes obtained using humanized mice. Considering this point, we mainly characterize the current conventional humanized mouse reconstituted only with HSCs and describe past achievements in this area, as well as the potential contributions of the humanized mouse models for the study of HIV pathogenesis and therapy. We also discuss the use of various technologies to solve the current problems. Humanized mice will contribute not only to the pre-clinical evaluation of anti-HIV regimens, but also to a deeper understanding of basic aspects of HIV biology.

## 1. Introduction

Mice reconstituted with human hematopoietic cells are called humanized mice and are applied as in vivo small animal models for the investigation of functional human immune cells and human-specific pathogens, including human immunodeficiency virus (HIV) [1,2,3,4]. Their advantages include the fact that: (i) almost all tissues, including the central nervous system (CNS), which is hardly accessible in living humans, are available at different stages of HIV infection; (ii) humanized mice can be maintained at lower cost than non-human primate (NHP) models; and (iii) unlike the NHP model which requires the modification of HIV genome, HIV can efficiently replicate in the humanized mice. There are several models of humanized mice using different mouse strains and different engraftment methods, and the history of humanized mice has been reviewed in detail elsewhere [2,3,4,5,6,7].

The common humanized mice currently in use largely represent two types of model. In the first, severely immunodeficient mice are transplanted with human CD34^+^ or CD133^+^ hematopoietic stem cells (HSC) typically sourced from umbilical cord blood (CB) or fetal liver (FL), although using FL-derived HSCs (FL-HSC) is more feasible than using CB-derived HSCs (CB-HSC) for the reconstitution of human hematopoietic cells in mucosal tissues such as the intestinal and genital tracts (see the review [5]). Representative mouse strains utilized for humanization are listed in Table 1. Humanized mouse models using non-obese diabetic (NOD)/SCID/IL2Rγ^null^ (NSG or NOG), NOD/Rag1^null^/IL2Rγ^null^ (NRG), and BALB/c/Rag2^null^/IL2Rγ^null^ (DKO/BRG) mice are called “current-generation models” [8]. Humanized mouse models using NOD/SCID/Jak3^null^ (NOJ) mice, which our research group has conventionally used, are also included in “current-generation models”, since these mice have a phenotype identical to that of NSG/NOG mice due to their deficiency of the IL-2Rγ downstream molecule Jak3 [9]. Furthermore, to improve immunological functions, several mouse strains expressing transgenic or knock-in molecules such as human leukocyte antigen (HLA), human cytokines, or murine thymic stromal lymphopoietin (TSLP) have been developed; humanized mice based on these mouse strains are called “next-generation models” [8]. The second model is bone marrow (BM)-liver-thymus (BLT) mice, which are transplanted with human FL and thymus, followed by irradiation, prior to transplantation with autologous HSCs. BLT mice are an excellent humanized mouse model in terms of HLA-restricted cellular immune responses. In BLT mice, human T cells can be educated in the autologous HLA-expressing thymus and HLA-restricted T cells can be elicited by infection or immunization [10,11,12].

Although FL-HSCs are now available from some vendors, the construction of BLT mice has been restricted in many countries except the United States and China due to the ethical and legal issues regarding aborted fetal sources. Even in the United States, the use of fetal sources is being restricted [13]. Therefore, non-BLT humanized mice, such as the former model described above, are preferentially used worldwide and will become even more important in the future. In this review, we mainly focus on non-BLT humanized mouse models and discuss the currently recognized limitations of humanized mouse models and their potential contributions to the study of HIV pathogenesis and therapy.

**Table 1:** Mouse strains for non-BLT humanized mice described in this review.

Generation	Strain	Genetic Manipulation	Original References or JAX ^a^ Stock Numbers
Current	NSG	*Il2Rγ* KO (complete) on NOD/SCID background	[14]
	NOG	*Il2Rγ* KO (truncated) on NOD/SCID background	[15]
	NOJ	*Jak3* KO on NOD/SCID background	[9]
	NRG	*Rag1* and *Il2Rγ* KO on NOD background	[16]
	DKO/BRG	*Rag2* and *Il2Rγ* KO on BALB/c background	[17]
Next	NSG-A2/HHD	*HLA-A2* and human *β_2_-microglobulin* Tg on NSG background	[18]
	NSG-DR4	*HLA-DR4* Tg on NSG background	029295
	NSG-Ab^0^DR4	*HLA-DR4* Tg and *mouse MHC-II* KO on NSG background	031566
	DRAG	*HLA-DR4* Tg on NRG background	[19]
	DRAGA	*HLA-A2* Tg on DRAG background	[20]
	BRGS	NOD *Sirpa* backcrossed to BRG background	[21,22]
	BRGST	mouse *Tslp* Tg on BRGS background	[23]
	NSG-SGM3	human *SCF*, *GM-CSF*, and *IL3* Tg on NSG background	[24]
	NSG-Quad	human *M*-*CSF* Tg on NSG-SGM3 background	[7]
	MISTRG	human *M-CSF*, *SCF*, *GM-CSF*, *IL3*, *thrombopoietin*, and *SIRPA* KI on DKO background	[25]
	SGR-15	human *IL15* and *SIRPA* KI on DKO background	[26]
	NSG hIL-7xhIL-15	human *IL7* and *IL15* KI on NSG background	[27]
	NSG-Tg(huIL6)	human *IL6* Tg on NSG background	[7]
	NOG-hIL-34	human *IL34* Tg on NOG background	[28]

^a^ The Jackson laboratory (Bar Harbor, ME). KI, knock-in; KO, knockout; Tg, transgenic.

## 2. Immunological Characteristics of Non-BLT Humanized Mice

Non-BLT humanized mice are conventionally constructed by the transplantation of HSCs into the livers of newborns or by the intravenous injection of HSCs into severely immunodeficient adult mice, in which the BM niche is disrupted by irradiation or busulfan administration [29,30]. G-CSF-mobilized blood/BM-derived HSCs were previously applied in humanized mice based on the established protocols for clinical HSC transplantation [31,32]. However, many current studies utilize CB- or FL-HSCs because of their superiority with respect to long-term engraftment and multipotency [6]. Since FL/BM-HSC transplantation into CB.17-*Prkdc^scid^* (SCID) mice was first attempted [33,34], a variety of immunodeficient mice have been developed, and the potential for the engraftment and differentiation of HSCs in them has been investigated [7,8,35].

In non-BLT humanized mice, T cells, B cells, and various innate immune cells are reconstituted in the BM, peripheral blood, and spleen [9,14,15,36]. However, the timing of the development of human hematopoietic cells differs between cell types. While B cells and myeloid cells develop early (within 8 weeks after HSC transplantation), T cells develop much later (approximately 12 weeks later), as observed in the peripheral blood [37,38,39,40]. Notably, CD4^+^ T cells develop more slowly than CD8^+^ T cells, but eventually end up massively outnumbering the latter and skew toward an activated effector memory phenotype, accounting for more than 60% of the CD4^+^ T cells 20 weeks after HSC transplantation [38]. In other words, the composition of CD4^+^ T cell subsets (naïve, central memory, and effector memory) changes over time in humanized mice. This is in contrast to the case in humans, since the effector memory subset, accounting for approximately 20% of CD4^+^ T cells in human peripheral blood, is maintained at a steady state [41]. As the effector memory subset potentially supports productive CCR5-tropic (R5) HIV-1 infection [38,42], the infectivity of R5 HIV-1 could be altered according to the age of the mouse after HSC transplantation [38].

The functional aspect of human immune cells in non-BLT humanized mice has been well described in many studies [6,7,35,43]. As human T cells developed in non-BLT humanized mice are educated depending on the mouse MHC, antigen-specific immune responses can hardly be induced by immature antigen-presenting cells that are differentiated from the same hematopoietic stem cells. Furthermore, the B cells also exhibit an immature phenotype, with impaired class switching and a low production of antibodies [6,23,44,45]. In addition, due to the lack of human cytokines, myeloid antigen-presenting cells such as dendritic cells (DC) and macrophages, as well as natural killer (NK) cells are poorly differentiated and maintain a low maturation and function status [44,46,47].

It has also been shown that lymphoid and myeloid progenitors are present in BM and contribute to the maintenance of lymphocyte and myeloid cells [9,48,49]. However, in non-BLT humanized mice, secondary lymphoid tissues other than the spleen are poorly developed. This is due to an IL-2 receptor γ-chain (IL-2Rγ) mutation in the host mouse, which leads to defective IL-7 signaling and the absence of lymphoid tissue inducer (LTi) cells [50]. The absence of LTi cells may contribute to the defective formation of lymph nodes and Peyer’s patches [50,51], the disorganization of follicular structures in lymph nodes [23], and a low level of human cell reconstitution in gut-associated lymphoid tissues [52,53]. Thus, whereas sufficient human CD45^+^ leukocytes are found in the peripheral blood and spleen, the distribution of these cells in other secondary lymphoid tissues is limited in non-BLT humanized mice [6,45].

## 3. Current Approaches to Overcoming Limitations of Non-BLT Humanized Mice

As mentioned above, non-BLT humanized mouse models still have the shortcomings of not only the insufficient functionality of CD4^+^ and CD8^+^ T cells but also the poor differentiation of myeloid lineage cells, such as monocytes, macrophages and DCs, all of which are involved in immune responses against HIV. In this section, we will discuss examples of non-BLT humanized mice (current-generation model) with additional treatments and also refer to the genetic manipulation of the host mice (next-generation model).

### 3.1. Modification of HSC Transplantation

In non-BLT humanized mice, the CD4^+^ T-cell reconstitution in the peripheral blood and spleen is sufficient; however, the reconstitution in mucosal tissue is very poor. To date, this shortcoming has only been overcome by transplanting fetal tissue or HSCs to construct humanized mice [5]. Recently, however, a fetal-tissue-independent model named CD34+T mice was developed [54]. This model was constructed by the intraperitoneal injection of autologous CD34^−^ cord blood mononuclear cells (CBMC) into CB-HSC-NRG mice 12 weeks after HSC transplantation, followed by administering four subcutaneous injections of recombinant human IL-7 on Days 0, 1, 2, and 7 after the CBMC injection. This resulted in high levels of T-cell reconstitution and mucosal HIV-1 transmission [54]. Another case where improved human immune responses can be achieved is in CB-HSC-NOG mice, which are constructed by directly transplanting CB-HSCs into BM (called IBMI-huNOG mice). In this mouse, stable T-cell numbers and B-cell-to-T-cell ratios are observed for 8 months after the HSC transplantation, and human T-cell leukemia virus type 1 (HTLV-1)-specific CD8^+^ cytotoxic T lymphocytes (CTL) and IgG antibodies are induced upon infection with HTLV-1 [55].

### 3.2. Supplementation of Human Cytokines

Exogenous cytokine administration has been attempted to induce myeloid cell differentiation in non-BLT humanized mice. In CB-HSC-NSG-A2/HHD mice, the development of human conventional DC type 1 (cDC1) and type 2 (cDC2) is enhanced by treatment with recombinant human FMS-like tyrosine kinase 3 ligand (FLT3L). Notably, these DCs differentiated from HLA-A2^+^ HSCs are able to prime CD8^+^ T cells in an antigen-specific, HLA-A2^+^-restricted manner [56]. An easier and less expensive alternative, “hydrodynamic gene delivery”, induces cytokines through the injection of plasmid DNA encoding human cytokine genes. In fact, the in vivo transfection of GM-CSF and IL-4 genes into CB-HSC-NSG mice enhances human DC differentiation, and, surprisingly, higher levels of antigen-specific IgG and CD4^+^ T cells are observed [44]. Our group also showed that the in vivo transfection of human FLT3L and GM-CSF genes into CB-HSC-NOJ mice not only enhanced the differentiation and maturation of human cDC1s and cDC2s [47] but could also induce the development of DC3s [57], a novel DC subset recently reported in humans [58,59,60], in the spleen and BM.

Furthermore, because of the essential role of IL-15 in the development of NK cells [46], the in vivo transfection of the human IL-15 gene by hydrodynamic gene delivery into CB-HSC-NSG mice enhances the development of human NK cells; the supplemental transfection of the human FLT3L gene further enhances this effect [61].

### 3.3. Transgenic or Knock-in Mice

Many transgenic mice have been established by introducing HLA genes into immunodeficient mice so that the education of human T cells in the thymus occurs through interaction with the same HLA instead of the mouse MHC. For example, in transgenic NSG mice (NSG-A2/HHD mice) that expressed the binding domain of HLA class I-A2 and human β_2_-microglobulin, functionally matured HLA-A2^+^ CB-HSCs-derived CD8^+^ T cells were developed [18]. As for HLA class II transgenic mice, HLA-DR4-expressing NSG strains (NSG-DR4 and NSG-Ab^0^DR4) and an NRG strain (NRG-DR4 (DRAG)) were developed (see the review [7]). In particular, it was reported that functional CD4^+^ and CD8^+^ T cells were differentiated and that CD4^+^ T cells were able to support the B-cell immunoglobulin class switching in HLA-DR^+^ CB-HSC-DRAG, as well as -DRAGA mice that express HLA-A2 on a DRAG background [19,20].

Instead of the transgenic expression of HLA, the overexpression of mTSLP in immunodeficient mice was shown to improve the immune response of T and B cells. BRGST mice [23] were independently established by crossing mTSLP transgenic mice [62] to BALB/c *Rag2*^−/−^ *IL2Rγ*^−/−^ *Sirpα^NOD^* mice (BRGS) [21]. In this mouse, the IL-7 signaling for the LTi cell differentiation pathway, which was attenuated by the IL2Rγ mutation, was complemented by mTSLP overexpression. As expected, CB-HSC-BRGST mice exhibited robust lymph node formation with compartmentalized human T and B cells. Accordingly, the antigen-specific responses were much improved by the development of mature B cells and IL-21-producing follicular helper T (Tfh) cells [23].

Improving the development of human myeloid lineage cells is another important issue. Many transgenic mice with human cytokine genes have been established. In particular, human stem cell factor (SCF), granulocyte macrophage colony-stimulating factor (GM-CSF), and IL-3 transgenic NSG (NSG-SGM3) mice exhibit a higher differentiation of human myeloid cells and regulatory CD4^+^ T (Treg) cells than NSG mice after CB-HSC transplantation [63]. However, the differentiation of human macrophages in CB-HSC-NSG-SGM3 mice remains poor. Recently, NSG-Quad mice were established by crossing human macrophage-colony stimulating factor (M-CSF) transgenic NSG mice with NSG-SGM3 mice, resulting in the enhanced differentiation of the CD14^+^ monocyte and macrophage population after CB-HSC transplantation [7]. Although not currently commercially available, DKO strain-based MISTRG mice were produced, in which the murine M-CSF, IL-3, GM-CSF, Sirp-α, and thrombopoietin genes were replaced by their human counterparts. They permit a long-term engraftment of CB-HSCs without irradiation and further differentiation of functional T, B, NK, and myeloid cells [25]. The developmental pathways from human blood monocytes to lung macrophages were recently revealed using CB-HSC-MISTRG mice [64]. Therefore, the differentiation of human myeloid cells in peripheral tissues can be reproduced in next-generation humanized mice. To further improve human NK cell development, human IL15 and Sirp-α knock-in SGR-15 mice (DKO background) [26] and human IL7 and IL15 knock-in NSG hIL-7xhIL-15 mice (NSG background) [27] were developed.

By modifying the cytokine environment of humanized mice, it is now possible to achieve the differentiation of functional human immune cells to some extent in humanized mice. The improved humanized mice as described above will be helpful for elucidating the relationship between in vivo HIV pathogenesis and various immune cells in local tissues.

## 4. Dynamics of Plasma Viral Load in HIV-Challenged Humanized Mice

Once HIV-1 has infected humans, the virus’ replication can be monitored by measuring plasma viral loads (VL). The two phases are observed during an initial HIV-1 infection in humans, the eclipse and burst phases. Described below are some examples of how the dynamic changes in HIV-1 replication occur in humanized mouse models.

### 4.1. From Eclipse to Burst Phase

In HIV-1-infected individuals, there is a virus-free period of approximately 10 days following the exposure of HIV-1, known as the eclipse phase. At the end of this phase, plasma HIV-1 can be detected and increases exponentially, peaking at 21–28 days post-infection [65,66,67]. In humanized mice, viremia is usually observed as early as 1 week after inoculation, regardless of the route of inoculation (intravenously, intraperitoneally, intrarectally, or intravaginally) and viral tropism (CCR5-tropic: R5; CXCR4-tropic: X4; or dual-tropic) [5,38,68,69,70,71,72,73,74,75,76,77,78]. It should be noted that plasma HIV-1 detected before 1 week may represent the inoculated viruses, because we observed that, when non-humanized NOJ mice were challenged with 2 to 50 ng of p24-measured amounts of HIV-1 intravenously or intraperitoneally, plasma HIV-1 was detected up to 5 days post-challenge for any HIV-1 dose inoculation (data not shown).

The time at which plasma viremia is observed in humanized mice is possibly affected by the viral dose at inoculation and the level of human chimerism [38,76]. Particularly, when we compared the plasma VLs at 1 week post-challenge between CB-HSC-NOJ mice that were either irradiated (IR^+^ hNOJ) or not (IR^−^ hNOJ) prior to human CB-HSC transplantation, the IR^+^ hNOJ mice showed significantly higher levels than the IR^−^ hNOJ mice did [38]. We used IR^+^ hNOJ mice at 10 weeks post-transplantation and IR^−^ hNOJ mice at ≥12 weeks post-transplantation. The IR^+^ hNOJ mice showed higher chimerism but a lower cell number of central and effector memory CD4^+^ T cells in the peripheral blood than the IR^−^ hNOJ mice [38], suggesting that cell populations other than CD4^+^ T cells contribute to the efficiency of early HIV-1 transmission.

Using FL-HSC-NOG mice, Sato et al. showed that the Vpu of HIV-1 contributed to efficient viremia during the early phase of infection by comparing wild-type and Vpu-deficient HIV-1 [79]. Interestingly, the authors also observed that most HIV-1-infected cells were in contact with the other infected cells in the spleen regardless of whether the infection was with wild-type or Vpu-deficient HIV-1, suggesting that cell-to-cell viral transmission frequently occurs in vivo [79]. DCs may play a central role in the virus’ dissemination in the body, since earlier in vitro studies demonstrated that DC–T cell contact efficiently transmits HIV-1 to CD4^+^ T cells, followed by the massive replication of HIV-1 in activated CD4^+^ T cells [80,81,82]. However, the efficiency of HIV-1 transmission by DC–T cell contact has not been fully evaluated in vivo, to date, in any non-BLT or BLT humanized mouse model, possibly due to the insufficient reconstitution of DCs; however, Murooka et al. showed, in an in vivo imaging analysis of HIV-1-infected BTL mice, that T cell–T cell contact via virological synapses efficiently spreads HIV-1 from infected to uninfected CD4^+^ T cells [83].

### 4.2. After Burst Phase

After the peak viremia, the VL decreases and reaches a stable level (known as the viral setpoint [67,84]), due to the development of virus-specific CTLs [85,86]. In HIV-1-infected humanized mice, the peak viremia is usually observed 2–6 weeks following systemic challenge. However, in contrast to that in humans, the viral setpoint is not obvious and the higher level of viremia continues as long as CD4^+^ T-cell hematopoiesis is maintained [70,75,87]. Whereas our group and Akkina’s group reported that R5 HIV-1 replicates more than X4 HIV-1 in humanized mice [68,72,73,77], some research groups have shown similar replication levels for R5 and X4 viruses [42,70,87]. This discrepancy might be due to the different experimental conditions used, including the type of HIV-1 strain and the status of the humanized mice. For example, we previously reported that aged CB-HSC-NOJ mice, in which activated memory CD4^+^ T cells are abundant, facilitate R5 HIV-1 replication (although X4 HIV-1 was not tested in this study) [38].

It is known that activated memory CD4^+^ T cells express more CCR5 but less CXCR4, resulting in them being more permissive to R5 HIV-1 but more restrictive for X4 HIV-1 [88,89]. Furthermore, when we investigated CB-HSC-NOJ mice that were singly or simultaneously infected with R5 HIV-1 and X4 HIV-1 using isogenic virus strains harboring DsRed or EGFP (R5: NL-AD8-D or X4: NL-E, respectively), we found that the X4 HIV-1 infection of CCR5^+^CD4^+^ T cells was suppressed in the presence of R5 HIV-1 [77]. As CCR5^+^CD4^+^ T cells would be a major producer of HIV-1 on the basis of their higher activation level, our previous finding would provide an insight into a mechanism by which R5 HIV-1 is dominantly isolated during the early stage of infection in patients [90,91,92], despite the fact that newly infected individuals have usually been exposed to a mixture of R5 and X4 viruses [93,94,95,96].

The persistent higher VLs in humanized mice may be due to the impaired or insufficient acquired immune responses. Even in BLT mice that can induce HIV-1-specific CTLs, it is not possible to reduce the plasma VL [97,98,99], apart from in those expressing HLA-B*57 (known as a protective HLA allele), which can reduce the plasma VL by about 10 times [100]. However, Gorantla et al. showed that the plasma VL increased following CD8^+^ T-cell depletion by the administration of anti-CD8 antibodies into HIV-1-infected CB-HSC-NSG mice [101], indicating that functional HIV-1-specific CTLs are induced to some extent. Furthermore, Palmer et al. showed that, when HIV-1-infected FL-HSC-DKO mice were treated with anti-programmed death-1 ligand-1 (PD-L1) antibodies to block the programmed death-1 (PD-1) pathway, the plasma VLs were decreased and the CD4^+^ T-cell counts were restored [102]. These findings suggest that there is room for the improvement of CTL functions even in non-BLT humanized mice.

## 5. Pathology in the Early Stage of HIV Infection

In the early stage of HIV-1 infection, several pathological and immunological events such as CD4^+^ T-cell depletion, the establishment of a virus reservoir, a cytokine storm, and immune activation are induced, and each is associated with disease progression [67,103,104,105,106]. One of the notable features of HIV pathogenesis is CD4^+^ T-cell depletion, which is observed during not only the late/AIDS stage but also the early stage of HIV-1 infection [67,107]; the level of CD4^+^ T-cell depletion is associated with the plasma VLs [108,109].

In some HIV-1-infected humanized mice, an obvious depletion of peripheral blood CD4^+^ T cells similar to that in HIV-1-infected individuals may not be observed because CD4^+^ T cells in CB-HSC-NSG mice continuously expand in the steady state at least up to 32 weeks after HSC transplantation [110]. Even if the CD4^+^ T-cell counts do not decrease in the peripheral blood, cell death is certainly induced in the tissues. We recently reported using CB-HSC-NOJ mice that, whereas a significant decrease in memory CD4^+^ T cells was observed 4 weeks after R5 HIV-1 challenge and that in naïve CD4^+^ T cells was observed 3 weeks after X4 HIV-1 challenge in the peripheral blood, cell death was induced as early as 3 days post-challenge with both R5 and X4 HIV-1 in the spleen [111]. Such an early induction of tissue CD4^+^ T-cell death by HIV-1 infection has also been observed in other humanized mice, such as FL-HSC-NOG [112], FL-HSC-NRG [113], and FL-HSC-DKO [113,114] mice.

### 5.1. Characteristics of CD4^+^ T-Cell Death

Earlier studies indicated that apoptosis is a central mechanism involved in the depletion of both HIV-1-infected and bystander CD4^+^ T cells [115,116,117,118,119]. Indeed, apoptotic cell death characterized by caspase-3 activation is preferentially induced in Treg cells during early HIV-1 infection, as observed in FL-HSC-NOG and FL-HSC-DKO mice [112,114], in a Vpr-dependent manner [112]. However, in humans, whether or not Treg cells are preferentially infected with HIV-1 is controversial (see reviews [120,121]). Therefore, these findings obtained from humanized mice may shed light on the Treg-associated human pathology.

It was reported that other types of non-apoptotic cell death, such as necroptosis and pyroptosis, are induced by HIV-1 infection in vitro using primary cells from the peripheral blood or the tonsils [122,123]. Therefore, our research group investigated whether such non-apoptotic cell death is induced in CD4^+^ T cells in vivo during the early stage of HIV-1 infection. We recently showed in CB-HSC-NOJ mice that non-apoptotic CD4^+^ T-cell death including caspace-1^+^ pyroptosis and phosphorylated mixed-lineage kinase domain-like protein (pMLKL)^+^ necroptosis was induced in the spleen at 3 days post-HIV-1 challenge when HIV-1 productive infection was less visible [111]. In addition, caspase-3/7^+^ apoptosis was not enhanced by either R5 or X4 HIV-1, but rather repressed in naïve CD4^+^ T cells when compared with that for mock infection [111]. Although we did not observe a significant repression of apoptosis within the CD45RA^-^ memory CD4^+^ T cell population [111], the induction of apoptosis by Treg cells, most of which do not express CD45RA in humanized mice [47,112] and were thus included in the memory population, might have offset this repression.

The mechanism involved remains to be clarified. However, it is interesting to consider the mechanism proposed by Lee et al., where the type I interferon (IFN-I)-mediated JAK-STAT signaling pathway induces the *Bcl-xL* anti-apoptotic gene and promotes pyroptosis by inhibiting apoptosis in human precancerous respiratory epithelial cells infected with influenza A virus [124]. It is known that IFN-I is induced during acute HIV-1 infections (the details are described below). However, it is apparent that IFN-I-mediated activation induces the apoptosis of CD4^+^ T cells during HIV-1 infection [125]. The mechanism that regulates the induction of apoptosis or pyroptosis during HIV-1 infection should be clarified in the future.

It was also reported that necroptosis is induced by both R5 and X4 HIV-1 in primary CD4^+^ T cells derived from the peripheral blood, although X4 HIV-1 induces necroptosis more efficiently than R5 HIV-1 does [123]. However, for a long time, it remained unclear whether necroptosis was induced in CD4^+^ T cells following HIV-1 infection in vivo until we showed it in CB-HSC-NOJ mice. We showed that pMLKL^+^ necroptosis could be induced by X4 HIV-1 but not R5 HIV-1, suggesting that naïve rather than memory CD4^+^ T cells are sensitive to necroptosis [111].

Pyroptosis and necroptosis are known forms of inflammatory programmed cell death and are considered to be responsible for the host defense against several pathogens, especially in early infection stages [126,127,128,129]. In HIV-1 infection, however, it seems that the high rates of CD4^+^ T-cell death are the consequence rather than the cause of immune activation [130]. On the other hand, pyroptosis and necroptosis are known to be induced by direct HIV-1-infection, and not in bystander cells [122,123]. We believe that humanized mice can show the beneficial (immunological) or detrimental (pathological) roles of cell death induced in the early stage of HIV-1 infection in vivo.

### 5.2. Immune Activation

HIV-1 infection triggers immune activation, and persistent immune activation is a fundamental driving force in disease progression [67,131]. A robust innate immune response mainly characterized as the upregulation of IFN-I and IFN-I stimulated genes accompanies early HIV-1 infection in patients [132]. The innate immune activation during early HIV-1 infection may promote HIV-1 replication, as evidenced by the fact that activated DCs can transmit the virus to CD4^+^ T cells and that the chemokines produced by plasmacytoid DCs (pDC) can recruit susceptible CD4^+^ T cells to the site of infection [67].

Even non-pathogenic simian immunodeficiency virus (SIV)-infected African natural hosts such as sooty mangabeys and African green monkeys also induce innate immune activation during early infection. However, this decreases during chronic infection, which is in contrast to the case in pathogenic SIV-infected Asian macaques [133,134]. Therefore, it suggests that innate immune activation per se is not necessarily detrimental. Rather, IFN-I contributes to protecting against early HIV-1 infection [135]. Indeed, an earlier study by Lapenta et al. using a classical humanized mouse model (human peripheral blood leukocytes (PBL)-transplanted SCID mice) demonstrated that treatment with IFN-I before HIV-1 challenge suppressed HIV-1 replication and preserved CD4^+^ T cells [136].

The current generation of humanized mouse models described by Li et al., using FL-HSC-DKO or -NRG mice, provide further significant insights in vivo; the depletion of pDCs by treatment with pDC-specific monoclonal antibodies resulted in the absence of plasma IFN-I and enhanced plasma HIV-1, indicating that pDCs are the critical producer of IFN-I and protect against viral infection during the early infection phase [113]. However, IFN-I is a double-edged sword: whereas it acts in a protective manner during early HIV-1 infection, it becomes pathogenic during chronic or persistent infection. Indeed, in combination antiretroviral therapy (cART)-suppressed, HIV-1-infected BLT mice, the blocking of the IFN-I pathway during the chronic or persistent infection phase results in the reversal of aberrant immune activation, reduced viral reservoirs, the restoration of CD8^+^ T-cell functions, and the restoration of CD4^+^ T-cell counts [137,138]. The IFN-I pathway was blocked in non-BLT humanized mice during the chronic or persistent infection phase without cART treatment, which restored CD4^+^ T-cell counts in the spleen but enhanced plasma HIV-1 [113]. However, this was not tested in the presence of cART treatment.

The loss of Treg cells may also be involved in the immune activation established in early HIV-1 infection [67,120,121]. In line with this, Sato et al. [112] showed using FL-HSC-NOG mice that the depletion of Treg cells by treatment with denileukin diftitox enhances CCR5 expression in memory CD4^+^ T cells and increases the slope of virus growth following HIV-1 infection, suggesting that immune activation due to the absence of Treg cells massively propagates HIV-1.

Due to the restricted availability of human clinical specimens, humanized mice are a convenient and important experimental animal model for the investigation of HIV pathogenesis, especially in the acute phase of infection. Taken together, although the immune responses in non-BLT conventional mice are limited, these mice recapitulate several significant aspects of HIV pathogenesis in humans, at least until plasma viremia reaches the peak level.

## 6. Pathology in the Gastrointestinal Tissues

CD4^+^ T cells in the gastrointestinal tissues are massively depleted prior to their loss in the peripheral blood during the early stage of HIV-1 and SIV infection, regardless of the route of virus transmission, because gastrointestinal CD4^+^ T cells are composed of high numbers of activated memory CCR5^+^CD4^+^ T cells [139,140,141]. In addition, peripheral CD4^+^ T cells expressing the gut-homing receptor α_4_β_7_ integrin can be efficiently infected with or bind to HIV-1 via α_4_β_7_ integrin-gp120 envelope protein interaction and may transmit viruses by cell-to-cell contact in the gastrointestinal tissues [142]. The massive HIV-1 infection of gastrointestinal CD4^+^ T cells leads to a weakened mucosal barrier, resulting in microbial translocation and persistent systemic immune activation during the chronic but not early stage of HIV-1 infection [143]. Th17 cells are a key player in intestinal barrier functions and are preferentially lost from the gastrointestinal tissues, but not the peripheral blood and the bronchoalveolar lavage, independently of direct virus infection [144]. Collectively, pathology in the gastrointestinal tissues sets the stage for HIV-1 disease progression to AIDS [130,145].

Therefore, it is important to know how precisely humanized mice can recapitulate HIV pathogenicity in human gastrointestinal tissues when applying humanized mice in pre-clinical trials. It has been shown that host IL-2Rγ is critical for the reconstitution of human hematopoietic cells in the gastrointestinal tissues of humanized mice [52]. Accordingly, the reconstituted levels of gastrointestinal human hematopoietic cells in non-BLT (current-generation) humanized mouse models, such as FL-HSC-NSG and -DKO mice, are far below those in BLT mice that are developed from NOD/SCID mice or even NSG mice [52]. However, a next-generation model using DRAG mice may overcome this limitation. In fact, a higher level of human hematopoietic cells can be reconstituted in not only the gastrointestinal but also the female reproductive tissues [69]. Interestingly, this mouse model further revealed that mucosal Tfh cells are highly permissive to HIV-1 [69]. In terms of Th17 cells, the existence was shown in human IL-6-transgenic NSG (NSG-Tg(huIL6)) mice transplanted with CB-HSCs [7]. We also showed that Foxp3^Low^ Th17-like cells are induced in the spleen when CB-HSC-NOJ mice are transfected in vivo with human *FLT3L* and *GM-CSF* genes [47]. However, the immunological role of gastrointestinal Th17 cells during HIV-1 infection has not been investigated in any humanized mouse model.

## 7. Pathology in the CNS

While cART has successfully extended the lifespans of patients, the issue of HIV-associated neurocognitive disorders (HAND) has emerged [146]. It is known that HIV-1 permissive cells such as microglial cells, macrophages, and CD4^+^ T cells exist in the CNS, and it has been suggested that ongoing inflammation triggered by HIV-1 infection in the CNS contributes to cognitive dysfunction [147,148]. However, the pathological mechanism leading to HAND has not been fully clarified. In addition, low levels of virus replication occur in the brain even during cART due to the limited drug penetrance into the CNS. Additionally, which types of HIV-1-permissive cells are truly HIV-1 producers or reservoirs is under debate, although microglial cells are the most likely [149].

Therefore, the humanized mouse model is expected to allow the investigation of HIV pathology in the CNS. Regarding this point, Garcia’s group used human myeloid-only humanized mice (MoM), which were constructed by transplanting FL-HSCs or CB-HSCs into NOD/SCID mice and in which human T cells were completely absent. They showed that the total number of human myeloid cells, including macrophages, increases in the brain following HIV-1 infection compared to that in uninfected MoM [150]. On the other hand, using T cell-only humanized mice (ToM), which were constructed by transplanting only FL and thymus but not HSCs into NSG mice and in which human B cells, NK cells, and myeloid cells were completely absent, they also showed that T cells establish and maintain HIV-1 infection in the brain [151]. These studies, however, did not describe microglial cells. HIV-1 infection is less likely to be achieved in the brains of humanized mice, as evidenced in a non-BLT humanized mouse model (FL-HSC-NSG mice) showing an almost undetectable level of HIV-1 DNA and RNA in the brain following HIV-1 challenge [152]. To overcome this limitation, Mathews et al. recently developed human IL-34 transgenic NOG (NOG-hIL-34) mice and showed significant numbers of human microglial cells in the brains of CB-HSC-NOG-hIL-34 mice compared to the control (CB-HSC-NOG) mice [28]. As a result, CB-HSC-NOG-hIL-34 mice are capable of robust HIV-1 infection in the brain, exhibiting a 1000–10,000 times higher VL in the brain than CB-HSC-NOG mice [28]. Collectively, a humanized mouse model using NOG-hIL-34 mice would currently appear to be the best model for HIV-1 neuropathogenesis.

## 8. Consideration of Suitable Mouse Models for Long-Term Analysis of HIV Infection

For the long-term analysis of HIV-1 infection, it would be ideal for the human cells that support HIV-1 infection to be maintained for as long as a normal mouse lifespan. Myeloablative irradiation is conventionally performed to augment the engraftment of donor HSCs in the recipient mice. However, in SCID mice, DNA repair is deficient, and they are sensitive to irradiation, resulting in shortened lifespans [2,38,87,153]. Therefore, our research group has conventionally constructed humanized NOJ mice without irradiation before HSC transplantation [47,57,77,111], recognizing that there is a decrease in chimerism, as indicated elsewhere [38,87]. To avoid such limitations, busulfan treatment is a useful alternative to irradiation for myeloablation [29,153,154,155,156,157], but it should be noted that higher doses of busulfan are toxic and fatal [153,156]. Another option is to use a non-SCID strain of mouse such as the NRG or DKO mouse, which is less sensitive to irradiation [2].

It is well known that the major obstacle to curing HIV-1 is the presence of persistently infected latent reservoirs. Several strategies, such as “kick-and-kill”, “block-and-lock”, and genome editing, combined with the augmentation of anti-HIV immunity, have been used for eliminating latently HIV-1-infected cells [158]. Since it is possible to mimic the in vivo situation of HIV-1 latency with oral ART in mice [159,160,161,162,163], such strategies have been evaluated by employing humanized mouse models. There are numerous latency studies using BLT mice, and the characteristics of the humanized mouse models in latency studies were recently discussed [164]. Although the BLT mouse model is highly informative, non-BLT mouse models also provide important knowledge in this field. We hereby highlight recent studies related to cure strategies utilizing non-BLT humanized mouse models in the following sections.

### 8.1. Broadly Cross-Reactive Neutralizing Antibodies

As in other viral infections, HIV-1-infected individuals develop neutralizing antibodies several months after the initial infection. The therapeutic potential of using neutralizing antibodies has been extensively studied, but the potency and breadth of broadly cross-reactive neutralizing antibodies (bNAb) have been limited (reviewed in [165]). Now, it is possible to identify a single B cell that produces bNAb in HIV-1-infected individuals and to analyze the precise structure of antibodies interacting with epitopes in the HIV-1 envelope protein. To date, various bNAbs with distinct epitopes have been identified, and some of them are already being investigated in clinical trials (reviewed in [166]). Humanized mouse models have been commonly used for these pre-clinical studies to design a cure strategy using bNAb therapy.

Although even the combination of five bNAbs with distinct epitopes is not enough to protect against HIV-1 infection [167], the contribution of potent bNAbs to the control of co-existing antibody-sensitive viruses is suggested in viremic controllers expressing HLA-B57*01 and HLA-B27*05 [168]. Schoofs et al. identified SF12 and SF12-related bNAbs that recognize a glycan-dominated epitope on the silent face of the HIV-1 envelope with a different angle [169]. The SF12 antibody showed broader recognition than the originally identified glycan-recognizing VRC-PG05 antibody, and HIV-1 was controlled by a triple mixture of bNAbs—SF12, 10-1074, and 3BNC117—in seven of eight CB-HSC-NRG mice during the observation period. By further searching for a more potent neutralizing antibody, Shcommers et al. recently isolated a novel bNAb with a near-universal breadth, 1-18 antibody, from a long-term non-progressor [170]. Interestingly, the 1-18 bNAb recognized the CD4 binding site as did other known bNAbs, such as VRC01 and 3BNC117 [165], but the unique feature of 1-18 bNAb was its contact with highly conserved gp120 residues and the increase in the buried surface on gp120. As expected, it potently suppressed HIV-1 replication and restricted HIV-1 escape in HIV-1-infected CB-HSC-NRG mice upon monotherapy. The 1-18 bNAb will be a promising candidate for combination bNAb therapy in humans.

The enhanced clearance of HIV-1-infected cells by a bNAb, 3BNC117, was previously confirmed in FL-HSC-NRG mice, and the importance of the Fcγ receptor-mediated immune response was suggested [171]. Non-neutralizing antibodies are now considered to be protective through FcR-mediated immune responses, such as the antibody-dependent cell cytotoxicity. In this context, Horwitz et al. using FL-HSC-NRG mice demonstrated that non-neutralizing antibodies alter the course of HIV-1 infection through FcR-mediated immune responses [172]. The protective efficacy of bNAb PGDM1400 was reported using CB-HSC-NSG mice [173]. Of note, the epitope of PGDM1400 antibody is similar to that of PG16 antibody, which was previously considered to be only partially protective in the BLT mouse model. It has been discussed that, in contrast to the systemic HIV-1 infection in the conventional humanized mouse model, the massive HIV-1 infection in the engrafted human thymi of BLT mice may reduce the effect of antibodies administered intraperitoneally. Thus, we need to carefully consider the modes of HIV-1 infection in different humanized mouse models.

Finally, to achieve the long, active persistence of these bNAbs, adeno-associated virus (AAV) gene-delivery systems have been developed [166]. The effect of the vector delivery of antibody may be further enhanced by selecting a potent antibody such as 1-18 bNAb, as described above. In line with this, a tandem-bispecific neutralizing antibody-expressing AAV was designed [174]. This therapy is protective even when starting 2 weeks after HIV-1 infection in hu-PBL-NSG mice. However, because the hu-PBL-NSG model is only suitable for short-term analysis, the duration of this effect and escape-mutation issues need to be further evaluated.

### 8.2. Gene Therapy

A sterilizing cure in HIV-1 infection would be possible if all the hematopoietic cells were replaced with uninfected or infection-resistant cells, as in the case of the “Berlin patient”, who received BM stem-cell transplantations from a genetically CCR5-defective donor [158]. Stem cell-based gene therapy to introduce anti-HIV T-cell receptor expression or chimeric antigen receptor (CAR)-T cells was employed using BLT mice and has been reviewed elsewhere [175]. Non-BLT humanized mice also serve as a suitable model for assessing the efficacy of the genetic manipulation of HSCs by utilizing an HIV-based lentiviral vector for the transfection of foreign genes into HSCs. For example, soluble CD4, an entry inhibitor [176], and microRNAs against CCR5 [177] were studied regarding the suppression of HIV-1 replication using FL- and CB-HSC-NSG mice, respectively.

Concerning bNAb therapy, genetically modified HSCs secreting the bNAbs PGT128 or VRC01 were transplanted into NSG mice. The long-term persistence of anti-HIV bNAb-secreting HSCs was achieved in all the lymphoid tissues, and HIV-1 replication was suppressed in PGT128 antibody-secreting but not in VRC01 antibody-secreting mice [178]. However, the antibody concentration of VRC01 was not high enough to be protective. Moreover, the issue of the emergence of escape mutants remains, as discussed above.

### 8.3. In Vivo Latency

When the replication of HIV-1 was suppressed by oral cART with three drugs (TDF: tenofovir; FTC: emtricitabine; and RAL: raltegravir), latency was established in the FL-HSC-NSG model, which recapitulates the important features of HIV-1 latency after cART in vivo [162]. Although the number of resting memory CD4^+^ T cells was low, latently infected cells could be obtained from various tissues of humanized mice and produce virus after PHA stimulation, as the authors expected. Moreover, Latinovic et al. showed that the combined administration of CCR5-targeting drugs, MVC (maraviroc) and rapamycin, and cART (TDF, FTC, and RAL) improves the suppression of productive HIV-1 infection and the preservation of CD4^+^ T cells more than cART alone in FL-HSC-NSG mice [75].

The infected human cells in the tissues of humanized mice are easily monitored by using a reporter HIV-1. In this context, the combination of different fluorescence-expressing X4 and R5 HIV-1s that we developed previously [179,180] is useful in the NOJ mice model, as described above. Llewellyn et al. utilized HA-expressing reporter HIV-1 to monitor infected cells in their latency study and revealed that the latent virus is enriched in PD-1- and TIGIT-positive exhausting CD4^+^ T cells in FL-HSC-NSG mice [161]. Ventura et al. developed nanoluciferase-expressing reporter HIV-1 with a sensitivity of as few as 30–50 infected cells by non-invasive bioluminescent imaging [163]. The further improvement of this technology may make it possible to obtain clear images of an initial virus rebound in tissue and cells in situ after the interruption of cART or through latent reactivation in humanized mice.

HIV-infected macrophages are known to be relatively resistant to HIV-1-induced cytopathic effects and to localize in various immune-privileged sites, including the CNS; thus, a very low level of HIV-1 production from this type of cell may contribute to the HIV-1 persistence under cART [98,164]. Of note, the latent HIV-1 in infected monocytic cells, but not in CD4^+^ T cells, was reactivated by a bromodomain inhibitor, I-BET151, in FL-HSC-NRG on cART, which indicates a distinct role of cyclin-dependent kinase 2 (CDK2) and CDK9 in these cells [160]. It also suggests that the HIV-1 infection in these cells differently contributes to the pathogenesis. In this context, because no viral RNA and DNA were detectable in macrophages isolated from cART-treated BLT mice, the MoM model was developed for the study of HIV-1-infected tissue macrophages [98], as described in Section 7. However, these mice completely lack human T cells and may not reflect the real situation in HIV-1-infected humans. To overcome the poor development of myeloid cells, humanized mice may need to be supplemented with human cytokines, as discussed in Section 3, for the simultaneous study of latent HIV-1 infection in CD4^+^ T cells and macrophages.

Finally, the genome-editing strategy using the CRISPR-Cas9 system combined with long-active slow-effective release ART (LASER ART) was tested in a latently HIV-1-infected FL-HSC-NSG model [159]. By the adoptive transfer of splenocytes and BM cells from treated to uninfected humanized mice, the authors showed that the elimination of latent HIV-1 is achievable only by the use of a combination of these therapies.

Thus, as a therapeutic point of view, various “proof-of-concept”-type studies are feasible and may provide further insights into an HIV cure strategy.

For the reader’s convenience, the important findings of HIV-1 pathogenesis and therapy using each non-BLT humanized mouse model discussed in this review are summarized in Table 2.

## 9. Concluding Remarks

It is desirable for humanized mice to properly recapitulate HIV-1 infection, pathogenesis, and immune responses in order to test prophylactic and therapeutic interventions in diseases along with HIV-1 infection in vivo. To overcome or improve limitations, the availability of human cytokines was tested by exogenous administration or in vivo transfection, and transgenic or knock-in mice were developed. However, limitations may often lead to the discovery of new insights when they can be represented as a suitable negative control. For example, the T cell-absent myeloid-only humanized mouse (MoM) model is capable of sustaining a constant level of HIV-1 production (although only a few HIV-1 strains can replicate), indicating a non-negligible target of macrophages for HIV-1 infection in vivo. Researchers should choose optimal humanized mouse models according to the purposes of their studies, taking into account the benefits and limitations of the model used, and should interpret their results carefully.

## Figures and Tables

**Table 2:** Summary of major findings of HIV-1 pathogenesis and therapy using non-BLT mice cited in this review.

Category (Section No.)	Major Findings	Mouse Strain	Source of HSC	HIV-1 Strain (Tropism)	References
Target cells of infection (4.1)	The central memory and/or effector memory subsets of CD4^+^ T cells are preferentially infected with HIV-1.	NOG	CB	JRCSF (R5)	[42]
		NOJ	CB	NL-AD8-D (R5)	[38]
Kinetics of plasma VL (4.2)	Higher level of plasma VL of R5 HIV-1 compared to X4 HIV-1.	DKO	FL	BaL (R5), NL4-3 (X4), NLENG1-IRES (X4)	[72,73]
		BALB/c-Rag1^−/−^γc^−/−^	FL	BaL (R5), NL4-3 (X4)	[68]
		NOJ	CB	NL-AD8-D (R5), NL-E (X4)	[77]
	Depletion of CD8^+^ T cells enhances plasma VL.	NSG	CB	ADA (R5)	[101]
	Blockage of PD-1 pathway decreases plasma VL and restores CD4^+^ T-cell counts.	DKO	FL	BaL (R5)	[102]
	Vpu contributes to the efficient virus spread. Cell-to-cell contact with infected cells occurs in the spleen independently of Vpu.	NOG	FL	AD8 (R5), AD8D*vpu* (R5)	[79]
Cell death (5.1)	HIV-1-infected Treg cells undergo apoptosis.	DKO	FL	R3A (dual)	[114]
	The loss of Treg cells increases the slope of HIV-1 growth.	NOG	FL	JRCSF (R5)	[112]
	Non-apoptotic CD4^+^ T-cell death, including pyroptosis and necroptosis, are induced during the early phase of HIV-1 infection.	NOJ	CB	NL-AD8 (R5), NL-AD8-D (R5), NL4-3 (X4), NL-E (X4)	[111]
Innate immune activation (5.2)	pDC depletion leads to the absence of plasma IFN-I and the enhanced plasma HIV-1 during the early infection phase.	DKO or NRG	FL	R3A (dual)	[113]
	Blocking of the IFN-I pathway during the chronic/persistent infection phase without cART treatment enhances plasma VL.				
Gastrointestinal tissues (6)	Tfh cells in the mucosal tissues are highly permissive to HIV-1.	DRAG	CB	US-1 (R5), BaL (R5)	[69]
CNS (7)	Macrophages can sustain HIV-1 replication in the absence of T cells in vivo. Human myeloid cells accumulate in the brain following HIV-1 infection.	NOD-SCID	FL or CB	ADA (R5), CHO40 (R5), CHO40-4013 env (R5)	[150]
	Introduction of human IL-34 induces significant numbers of microglial cells and is capable of robust HIV-1 infection in the brain.	NOG-IL-34	CB	ADA (R5)	[28]
Antibody therapy (8.1)	The SF12 bNAb suppresses plasma VL and exerts strong selective pressure on HIV-1.	NRG	CB	YU2 (R5)	[169]
	The 1-18 bNAb suppresses plasma VL and restricts HIV-1 escape.	NRG	CB	YU2 (R5)	[170]
	The PGDM1400 bNAb provides protection against HIV-1 challenge.	NSG	CB	JRCSF (R5)	[173]
	FcR-mediated immune responses contribute to the clearance of HIV-1-infected cells.	NRG	FL	YU2 (R5)	[171]
				YU2 and its derivatives (R5)	[172]
Gene therapy (8.2)	Humanized mice reconstituted with soluble CD4 gene-transduced HSCs reduce plasma VL over time.	NSG	CB	BaL (R5)	[176]
	Humanized mice reconstituted with HSCs expressing microRNAs against CCR5 decrease plasma VL over months.		CB	YU2 (R5), JRCSF (R5)	[177]
	Humanized mice reconstituted with PGT128 bNAb genes-transduced HSCs decrease plasma VL.		FL	BaL (R5)	[178]
Latency (8.3)	Latent infection models are achieved by treatment with cART or the combined administration of CCR5-targeting drugs.	NSG	FL	BaL (R5)	[162]
			CB		[75]
	Using reporter HIV-1 enables to sensitively detect latent virus, which is enriched in PD-1^+^ and TIGIT^+^ CD4^+^ T cells.	NSG	FL	NL4-3-HA (X4)	[161]
	Longitudinal non-invasive bioluminescent imaging of HIV-1 infection dynamics using nanoluciferase-expressing reporter HIV-1 can sensitively detect infected cells following cART withdrawal.	NSG	FL	Q23.BG505.Nluc* (R5)	[163]
	Latent HIV-1 in infected monocytes but not in CD4^+^ T cells is reactivated by a bromodomain inhibitor.	NRG	FL	JRCSF (R5)	[160]
	The elimination of latent HIV is achievable by a combination of the CRISPER-Cas9 system and long-active slow-effective release ART.	NSG	FL	NL4-3 (X4)	[159]

## Data Availability

No new data were created or analyzed in this study. Data sharing is not applicable to this article.
